# Quantifying systemic congestion with Point-Of-Care ultrasound: development of the venous excess ultrasound grading system

**DOI:** 10.1186/s13089-020-00163-w

**Published:** 2020-04-09

**Authors:** William Beaubien-Souligny, Philippe Rola, Korbin Haycock, Josée Bouchard, Yoan Lamarche, Rory Spiegel, André Y. Denault

**Affiliations:** 1 Department of Anesthesiology and Intensive Care, Montreal Heart Institute, Université de Montréal, Montreal, QC Canada; 2 Division of Nephrology, Centre Hospitalier de l’Université de Montreal, 1000, Rue St-Denis, Montreal, QC H2X 0C1 Canada; 3 Division of Intensive Care, Santa Cabrini Hospital, Montreal, QC Canada; 4 Department of Emergency Medicine, Loma Linda University School of Medicine, Loma Linda, CA 92354 USA; 5 Division of Nephrology, Hôpital Sacré-Cœur de Montréal, Montreal, QC Canada; 6 Department of Surgery and Critical Care, Montreal Heart Institute, Université de Montréal, Montréal, QC Canada; 7 Departments of Critical Care and Emergency Medicine, Washington Hospital Center, Georgetown University, Washington, DC USA; 8 Division of Intensive Care, Centre Hospitalier de L’Université de Montreal, Montreal, QC Canada

**Keywords:** Acute kidney injury, Cardiac surgery, Point-Of-Care ultrasound, Venous congestion, Fluid balance

## Abstract

**Background:**

Organ congestion is a mediator of adverse outcomes in critically ill patients. Point-Of-Care ultrasound (POCUS) is widely available and could enable clinicians to detect signs of venous congestion at the bedside. The aim of this study was to develop several grading system prototypes using POCUS and to determine their respective ability to predict acute kidney injury (AKI) after cardiac surgery. This is a post-hoc analysis of a single-center prospective study in 145 patients undergoing cardiac surgery for which repeated daily measurements of hepatic, portal, intra-renal vein Doppler and inferior vena cava (IVC) ultrasound were performed during the first 72 h after surgery. Five prototypes of venous excess ultrasound (VExUS) grading system combining multiple ultrasound markers were developed.

**Results:**

The association between each score and AKI was assessed using time-dependant Cox models as well as conventional performance measures of diagnostic testing. A total of 706 ultrasound assessments were analyzed. We found that defining severe venous congestion as the presence of severe flow abnormalities in multiple Doppler patterns with a dilated IVC (≥ 2 cm) showed the strongest association with the development of subsequent AKI compared with other combinations (HR: 3.69 CI 1.65–8.24 *p* = 0.001). The association remained significant after adjustment for baseline risk of AKI and vasopressor/inotropic support (HR: 2.82 CI 1.21–6.55 *p* = 0.02). Furthermore, this severe VExUS grade offered a useful positive likelihood ratio (+LR: 6.37 CI 2.19–18.50) when detected at ICU admission, which outperformed central venous pressure measurements.

**Conclusions:**

The combination of multiple POCUS markers may identify clinically significant venous congestion.

## Background

Hemodynamic management in critically ill patients has traditionally focused on maintaining adequate cardiac output and arterial blood pressure by relying on fluid administration and vasopressor/inotropic support [1, 2]. However, organ perfusion is affected by other important factors [3, 4]. Among them, the venous pressure is often overlooked as a hemodynamic parameter that may be of critical importance. The development of clinically significant organ congestion is susceptible to occur in patients with right ventricular failure or pulmonary hypertension, and in patients with fluid overload. These contributors are likely to be synergistic in critically ill patients, particularly when renal dysfunction aggravates fluid retention. A reduction of the arteriovenous gradient across vital organs may hamper adequate perfusion [5]. This phenomenon may be worsened with the development of interstitial edema after prolonged elevation of capillary hydrostatic pressure in the context of a dysfunctional endothelial barrier [4]. In encapsulated organs such as the kidney and the brain, interstitial edema may result in a rapid elevation in interstitial pressure, which then decreases organ blood flow [6, 7]. Furthermore, interstitial edema is hypothesized to impair tissue oxygenation by increasing the diffusion distances within the interstitium [8].

An exceedingly challenging aspect of hemodynamic evaluation is determining what represents clinically significant venous hypertension. Central venous pressure (CVP) measurements performed in the intensive care unit (ICU) remain invasive and are subject to important measurement errors even among experienced operators [9]. Furthermore, while higher CVP has been associated with complications in multiple settings [10–12], it remains unclear what level of CVP is deleterious and may be considered a trigger for intervention [13]. Other metrics such as cumulative fluid balance, weight variations and physical examination for peripheral edema, each have important limitations and may not be proportional to systemic venous pressure [14–16].

Point-Of-Care ultrasound (POCUS) enables the clinician to visualize the vascular anatomy and assess blood velocity using Doppler imaging. Within any venous vascular system, the additional volume associated with congestive factors will eventually reach the upper limits of the systemic venous capacitance, causing a rapid rise in venous pressures. Several markers of the high pressures associated with this congestive process have been proposed including the assessment of large veins (vena cava, internal jugular) as well as detecting abnormal venous waveforms suggestive that the limit of the systemic venous compliance in the portal vein, hepatic veins and intra-renal veins [17]. All of these markers have been associated with adverse consequences of venous hypertension, both in acute and chronic settings [18–21]. However, they also all have significant limitations that may hamper their clinical usefulness when interpreted in isolation [22–24]. It is likely that considering a combination of these ultrasonographic features may increase the clinical usefulness of POCUS for the purpose of detecting significant venous congestion.

The primary objective of the exploratory study was to develop a prototypical Venous Excess Ultrasound (VExUS) grading system of the severity of venous congestion, and to validate its potential clinical value in predicting the occurrence of acute kidney injury (AKI) after cardiac surgery using existing data from a recent prospective cohort study. The secondary objective was to compare its clinical usefulness to CVP measurements.

## Methods

### Study design and participants

This is a post-hoc analysis of data collected during a prospective cohort study at a tertiary cardiac surgery center from August 2016 to July 2017 (NCT02831907) [21]. Written consent was obtained for all patients and the project was approved by the Montreal Heart Institute Ethics Committee (2016-1946).

Non-critically ill patients 18 years and older undergoing cardiac surgery with the use of cardiopulmonary bypass were eligible to participate. Complete inclusion and exclusion criteria have been previously described [21]. Notably, patients with critical illness, AKI or delirium before surgery were excluded as well as patients with conditions that may have interfered with portal Doppler assessment (cirrhosis, portal thrombosis) and patients with severe chronic kidney disease (estimated glomerular filtration rate < 15 mL/min per 1.73 m^2^ calculated using the Modified Diet in Renal Disease formula [25]) or dialysis.

### Ultrasound assessment

All patients underwent repeated POCUS assessment the day before surgery, at ICU admission after surgery and daily from post-operative days 1 to 3. Each ultrasound assessment consisted of hepatic vein Doppler, portal vein Doppler, intra-renal venous Doppler and inferior vena cava (IVC) ultrasound. The complete method for hepatic, portal and renal Doppler assessment has been previously published [21]. The assessments were performed with concurrent electrocardiogram tracing to adequately identify the hepatic waveform phases during the cardiac cycle. Inter-observer variability for identifying portal and intra-renal vein Doppler patterns were good, as previously reported [21].

The IVC diameter was measured in its intra-hepatic portion at 2 cm of the junction with the hepatic veins using a longitudinal view from a sub-xiphoid position [26]. When the sub-xiphoid window was not appropriate the probe was moved laterally to the right side of the body, over the liver, until an adequate view was achieved. The maximal diameter during the respiratory cycle was measured.

### Development of VExUS grading system prototypes

**Fig. 1:**
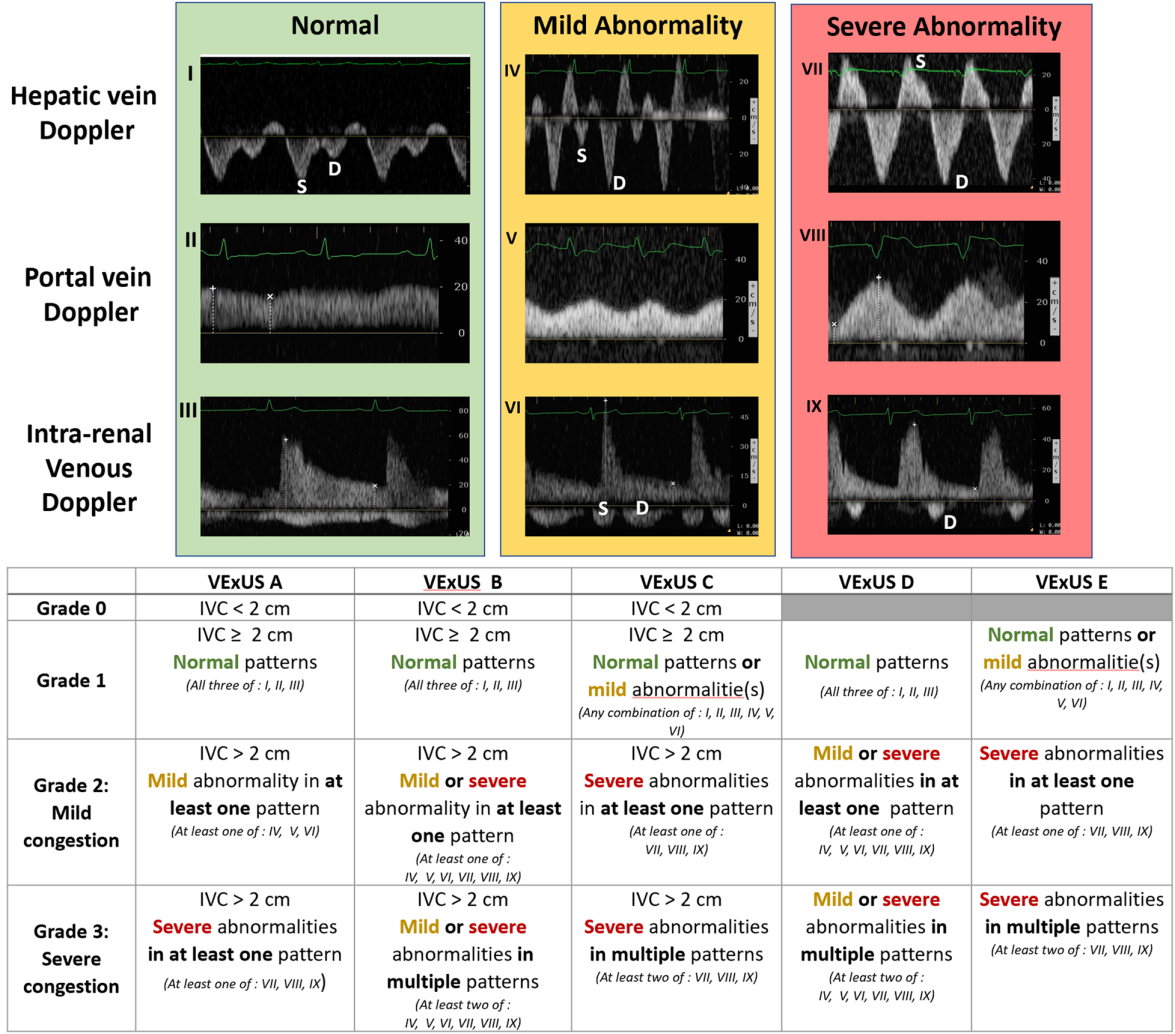
The Venous Excess UltraSound (VExUS) grading system prototypes combining inferior vena cava (IVC) diameter and venous Doppler waveform of the portal, hepatic and interlobular renal veins. Hepatic Doppler is considered mildly abnormal when the systolic (S) component is lower in magnitude than the diastolic (D) component, but still toward the liver while it is considered severely abnormal when the S component is reversed (toward the heart). Portal Doppler is considered mildly abnormal when a variation in the velocities during the cardiac cycle of 30 to < 50% are observed, while is considered severely abnormal when a variation of ≥ 50% is seen. Intra-renal venous Doppler is considered mildly abnormal when it is discontinuous with a systolic (S) and diastolic (D) phase, while is it considered severely abnormal when it is discontinuous with only a diastolic phase seen during the cardiac cycle

Based on an original concept (P.R.), a multidisciplinary team composed of intensivists (A.D., P.R.), anesthesiologist (A.D.), emergency physicians (R.S., K.H.) and nephrologist (W.B.S) developed five VExUS grading system prototypes based on the severity of venous ultrasonographic markers (Fig. 1). For the hepatic vein Doppler, a systolic phase was of lesser amplitude than the diastolic phase but toward the liver was considered mild while the presence of a reversed systolic phase was considered severe [17, 27]. For the portal vein Doppler, a pulsatility fraction (PF) of 30–49% was considered mild while a PF > 50% was considered severe based on the previous studies [21, 28, 29]. For the intra-renal venous Doppler, a discontinuous pattern with a systolic and a diastolic phase was considered mild while a discontinuous pattern with only a diastolic phase was considered severe [18, 21]. The prototype grading systems were named VExUS “A” through “E” (Fig. 1) with multiple grades within each grading system. The VExUS score was determined for all patients and for all timepoints. Overall, echographic variables were > 95% complete within the dataset. In the case of a missing value for an echographic marker, the last known value for this marker for the patient (i.e., assessment performed the previous day) was imputed.

### Clinical data collection

Demographic, baseline information, cumulative fluid balance information, hemodynamic parameters at the time of assessment and outcomes during hospitalization were all collected prospectively during the study as previously published [21]. The presence of left and right systolic ventricular dysfunction on trans-esophageal echocardiography performed by the attending anesthesiologist before cardiopulmonary bypass was also collected. Left ventricular dysfunction was defined as a left ventricular ejection fraction (LVEF) ≤ 40%, and right systolic ventricular dysfunction was defined by either tricuspid annular plane systolic excursion < 16 mm or right ventricular area of change < 35% [27]. Mean CVP measurements were obtained using a jugular central venous catheter. Measurements were noted before POCUS assessment at ICU admission and daily if the central line was still in place at the time of POCUS assessment. The mean CVP values were noted after verifying the position of the pressure transducer in relation with the patient. The height of the bed was adjusted if needed to insure proper positioning at the level of the right atrium (intersection between mid-axillary line and fourth intercostal space). N-terminal pro-beta natriuretic peptide (NT-pro-BNP) was also measured prospectively before surgery and on the morning of post-operative days 1, 2 and 3. The European System Operative Score Risk Evaluation score (EuroSCORE II) was calculated, as well as a validated risk score by Birnie et al. for the prediction of AKI in cardiac surgery patients based on pre-operative characteristics and validated for the Kidney Disease: Improving Global Outcomes (KDIGO) criteria [30]. During the post-operative period, AKI was defined by the KDIGO criteria as an increase of serum creatinine > 26 μmol/L within a 48‐h period or 50% from baseline creatinine within a week from cardiac surgery [31]. Vasopressors (norepinephrine, vasopressin, dopamine) and inotrope (epinephrine, milrinone, dobutamine) use was noted at the time of ultrasound assessment. The vasopressor–inotrope score (VIS) was calculated to estimate the degree of pharmacologic support at the time of ultrasound assessment [32, 33]. The VIS was calculated using the following formula: VIS = dopamine dose (μg/kg/min) + dobutamine dose (μg/kg/min) + (100 * epinephrine dose (μg/kg/min)) + (10 * milrinone dose (μg/kg/min)) + (10,000 * vasopressin dose (U/kg/min)) + (100 * norepinephrine dose (μg/kg/min)) [32, 34].

### Data analysis

Results are presented in number (%) for dichotomous variables and in mean ± standard deviation (SD) or median and interquartile range (IQR) for continuous variables, where appropriate. Comparisons between two groups for continuous variables were done using Student *t*-test or Mann–Whitney *U* test, as appropriate, and comparison between two groups for categorical variables was done using Chi squared test. The prevalence of each VExUS grade was presented as descriptive data for each timepoint. The association between the VExUS grades and the risk of new‐onset of AKI was assessed using a Cox proportional hazards model with the VExUS grades considered as segmented time‐dependent covariates. After identifying the VExUS grade most associated with AKI in univariable analysis, multivariable Cox regression was performed. A first model was constructed by including the pre-operative risk of AKI as performed by Birnie et al. [30] as an a priori covariate. This score included age, sex, body mass index, smoker status, New York Heart Association functional status, diabetes, peripheral vascular disease, chronic hypertension, hemoglobin level, renal function, recent coronary angiogram, triple vessel disease, operative priority and procedure type. In addition, a second model was created by adding the VIS as a segmented time‐dependent covariate to the first model. As a sensitivity analysis, other multivariable models including CBP duration and cardiac output measured at the end of surgery were performed. Results are presented as hazard ratio (HR) with 95% confidence intervals (CI).

The sensitivity and specificity as well as the positive likelihood ratio (+LR) and the negative likelihood ratio (−LR) of the different VExUS grades assessed at ICU admission to predict AKI after cardiac surgery were presented. The same analysis was performed using different cut-offs of CVP (≥ 8, ≥ 10, ≥ 12 and ≥ 14 mmHg), as well as individual ultrasound findings included in the VExUS grading systems. The CVP cut-off used was chosen based on current literature and expert opinion [10, 11, 13, 35]. Results are presented with 95% CI. Leaf plots were created to visually compare the diagnostic performance of grade 3 of the VExUS C grading system and CVP ≥ 12 mmHg [36, 37]. Leaf plots were generated using an online tool [38]. As a supplementary analysis, specificity of grade 3 of the VExUS C grading system was compared to other variables using exact McNemar test for paired nominal data.

The association between the VExUS grading system prototypes and commonly used markers of venous congestion (cumulative fluid balance, NT-pro-BNP and CVP) and VIS was first assessed using generalized estimating equation models using a robust estimator for the covariance matrix and an exchangeable structure for the working correlation matrix was used. This type of analysis accounts for the repeated measures’ design, implying that the sample was not independent. In addition to the VExUS grades, the time of assessment (4 timepoints: Day 0 to Day 3) was included as a factor in the analysis and the interaction between the studied variable and the time of ultrasound assessment was tested. We found multiple significant interactions (*p* < 0.05) with the time of assessment. Consequently, we presented the association for each timepoint and for each VExUS grade. The difference between the different grades was assessed using one-way ANOVA or Kruskall–Wallis test, where appropriate depending on the distribution of data. In the presence of a significant result (*p* < 0.05), multiple post-hoc pairwise comparisons with Bonferroni correction were performed and significant results were presented. Statistical tests were performed in SPSS version 24 (IBM, Armonk, New York, USA).

## Results

Complete data from all 145 participants included in the original prospective study were included in the analysis. Baseline characteristics of patients have been previously published [21] and a summary is available in Additional file 1: Table S1. The median age was 66 ± 13 years and the median risk of complications based on pre-operative characteristics according to the EuroSCORE II was 2.96% (1.70; 4.79%). Known heart failure with reduced ejection fraction (LVEF ≤ 40%) was present in 31 patients (21.4%) and stage III chronic kidney disease or higher (corresponding to an eGFR < 60 mL/min/1.73 m^2^) was present in 37 patients (25.5%). At the start of the surgery before cardiopulmonary bypass, trans-esophageal echocardiography revealed low LVEF in 37 patients (25.5%) and systolic right ventricular dysfunction in 18 patients (12.4%). After surgery, 49 patients (33.8%) developed AKI but no patients received renal replacement therapy.

**Fig. 2:**
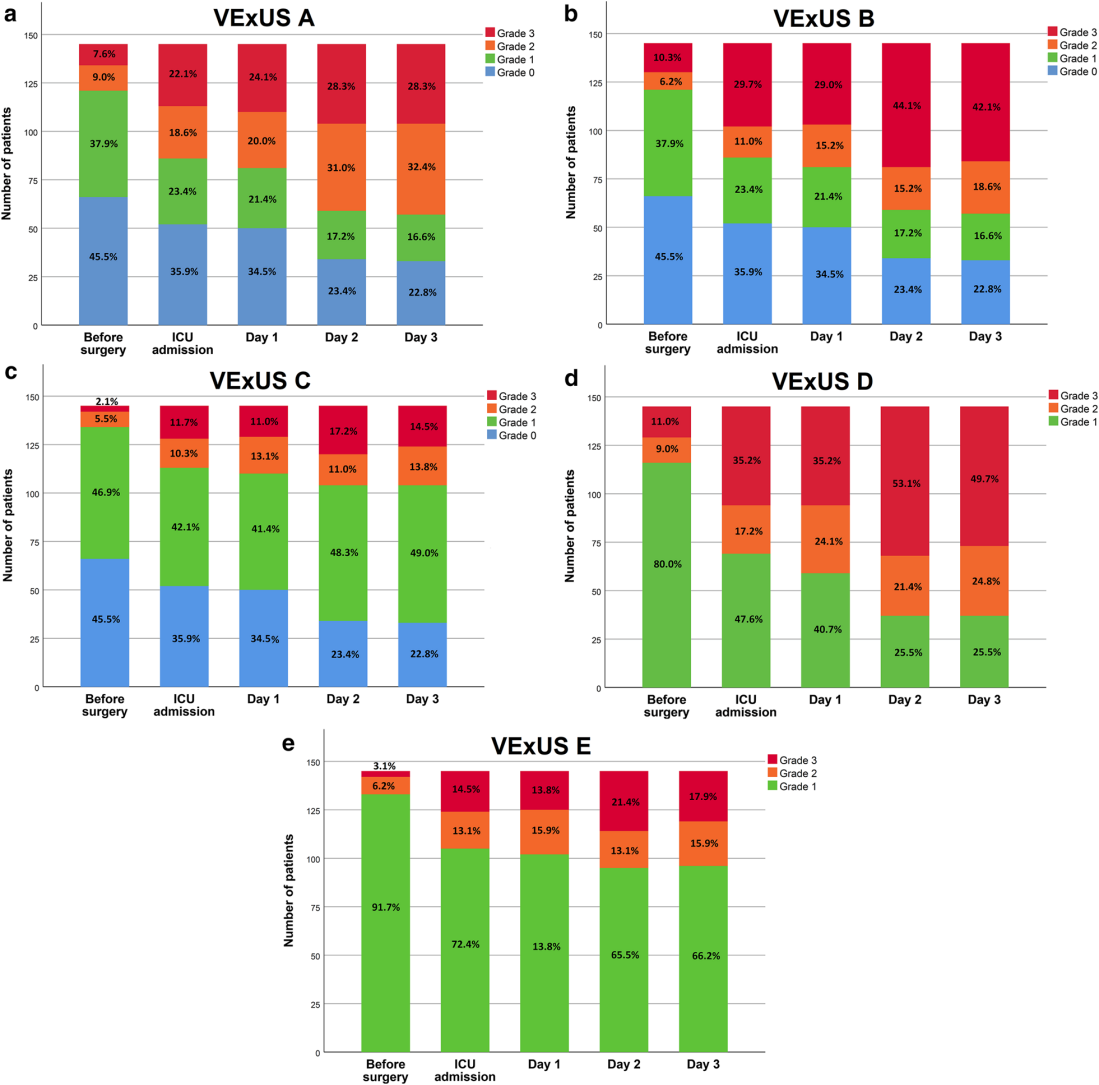
Distribution of Venous Excess UltraSound (VExUS) grading system prototypes **a**, **b**, **c**, **d** and **e** in the perioperative period in 145 patients undergoing cardiac surgery

The complete distributions of all VExUS grades (0–3) according to candidate grading systems (A–E) during the peri-operative period are presented in Fig. 2. Severe venous congestion (Grade 3) was less often diagnosed with grading systems requiring the presence of severe flow abnormalities in multiple Doppler patterns (VExUS grading systems C and E) compared with grading systems requiring at least one severe Doppler finding (VExUS grading system A) or the combination of mild and severe Doppler findings (VExUS grading systems B or D). Compared with scores which did not consider IVC measurements (VExUS grading systems D and E), corresponding grading systems which included IVC measurements (VExUS grading systems B and C) identified less patients as having severe congestion (Grade 3). Before surgery, EuroSCORE II was different for all VExUS grading systems, with more severe VExUS grades being associated with a higher pre-operative risk assessment (Additional file 1: Table S2).

In the post-operative period, the presence of severe congestion (Grade 3) was associated with the development of subsequent AKI for all VExUS grading systems (Table 1). Severe congestion (Grade 3) defined by the VExUS C grading system was the most strongly associated with AKI (HR 3.69, CI 1.65–8.24, *p* = 0.001). After adjustment for the baseline risk of AKI and pharmacologic support (VIS), severe congestion (Grade 3) defined by the VExUS C grading system remained associated with the subsequent development of AKI after surgery (HR 2.82, CI 1.21–6.55 *p* = 0.02) (Table 2). Adjustment with cardiac output measured at the end of surgery and cardiopulmonary bypass duration yielded similar results (Additional file 1: Table S3). As for moderate congestion (Grade 2), only the VExUS C grading system was associated with the development of AKI (HR 2.65, CI 1.07–6.60 *p* = 0.036).

**Table 1:** Association between Venous Excess UltraSound (VExUS) grading system prototypes and the risk of acute kidney injury in the post-operative period

Grading system	Grade	HR	CI	*p* values
VExUS A	0	Reference category		
	1	1.10	0.44–2.73	0.84
	2	1.41	0.58–3.43	0.44
	3	3.21	1.55–6.67	0.002
VExUS B	0	Reference category		
	1	1.10	0.44–2.74	0.84
	2	2.11	0.85–2.74	0.11
	3	2.43	1.18–5.02	0.02
VExUS C	0	Reference category		
	1	1.25	0.58–2.66	0.57
	2	2.65	1.07–6.60	0.036
	3	3.69	1.65–8.24	0.001
VExUS D	0	Reference category		
	1	1.71	0.78–3.74	0.18
	2	1.95	1.02–3.75	0.045
VExUS E	0	Reference category		
	1	1.72	0.78–3.80	0.18
	2	2.68	1.41–5.12	0.003

Proportional hazard regression models (Cox) with VExUS grading systems considered as time varying covariates (i.e., VExUS grade at day 0 is used to predict acute kidney injury (AKI) at day 1, VExUS grade at day 1 is used for AKI at day 2 and so on). *HR* hazard ratio, *CI 95%* confidence intervals)

**Table 2:** Multivariable proportional hazards models to predict acute kidney injury in 145 patients after cardiac surgery using the Venous EXcess UltraSound (VExUS) C grading system

	Crude hazard ratio			Model 1			Model 2		
				Adjusted hazard ratio^a^			Adjusted hazard ratio^b^		
	HR	CI	*p*	HR	CI	*p*	HR	CI	*p*
VExUS C Grade 0	Reference category			Reference category			Reference category		
VExUS C Grade 1	1.25	0.58–2.66	0.57	1.13	0.52–2.43	0.76	1.13	0.52–2.43	0.76
VExUS C Grade 2	2.65	1.07–6.60	0.036	2.31	0.92–5.80	0.074	2.32	0.92–5.83	0.073
VExUS C Grade 3	3.69	1.65–8.24	0.001	2.83	1.22–6.55	0.015	2.82	1.21–6.55	0.016
Pre-operative AKI risk score [30]	1.02	1.01–1.04	0.001	1.02	1.003–1.03	0.019	1.02	1.003–1.033	0.02
Vasopressor–inotrope score	1.01	0.99–1.03	0.51				1.001	0.98–1.03	0.93

Multivariable proportional hazard regression model (Cox) with the VExUS grade considered as a time-varying covariate (i.e., VExUS grade at day 0 is used to predict AKI at day 1, VExUS grade at day 1 is used for AKI at day 2 and so on). *HR* hazard ratio, *CI 95%* confidence intervals

^a^Variables included in the multivariate model were the VExUS C grade (segmented time-dependant) and pre-operative AKI risk score [30]

^b^Variables included in the multivariate model were the VExUS C grade (segmented time-dependant), vasopressor–inotrope score (segmented time-dependant) and pre-operative AKI risk score [30]

**Fig. 3:**
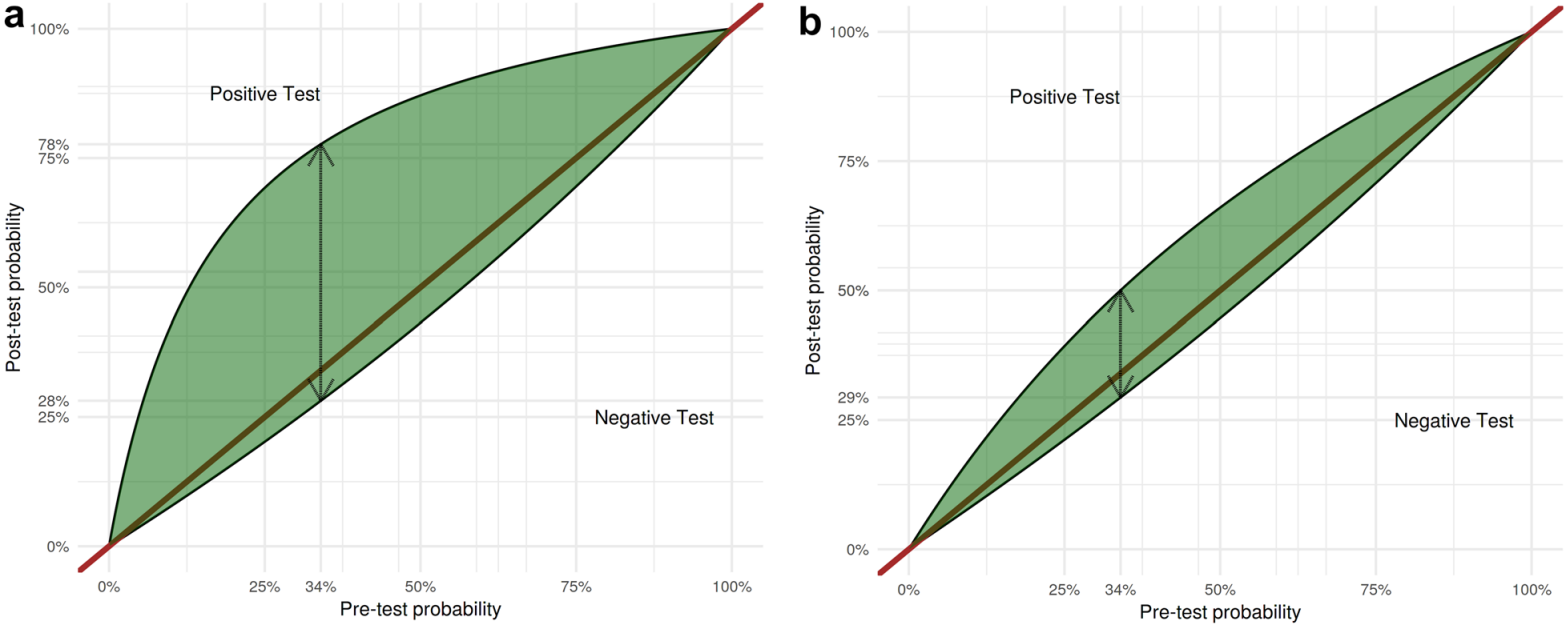
Leaf plots displaying the relationship between the assumed pre-test probability (on the *x*-axis) and the post-test probability (on the *y*-axis) of acute kidney injury (AKI) for the following cut-off: **a** severe congestion (Grade 3) defined by the VExUS C grading system and **b** central venous pressure of ≥ 12 mmHg. The upper half part of the curve indicates the post-test probability in case of a positive result while the lower half indicated is for a negative test result. The dashed double-sided arrow indicated the test performance considering the incidence of acute kidney injury (pre-rest probability) within the studied cohort (33.8%)

While only considering the assessment performed at ICU admission after surgery, severe congestion (Grade 3) defined by the VExUS C grading system had a high specificity (96% CI 89–99%) but low sensitivity (27% CI 15–41%) for the development of subsequent AKI resulting in a moderate +LR of 6.37 (CI 2.19–18.5) which surpassed the performance of other grading systems (Table 3). All VExUS grading systems had a low sensitivity (< 75%) resulting in poor − LR (> 0.5). A low CVP cut-off (≥ 8 mmHg) had a moderately useful − LR of 0.49 (CI 0.28–0.86). However, none of the studied CVP cut-offs or individual ultrasound markers outperformed grade 3 of the VExUS C grading system with respect to the +LR. The performance of VExUS C grade 3 and CVP ≥ 12 mmHg is represented graphically in Fig. 3. The specificity of grade 3 of the VExUS C grading system was statistically better than all other candidate variables except severe portal vein pulsatility, severe alteration of intra-renal venous flow and VExUS E grade 3 (see Additional file 1: Table S4).

**Table 3:** Performance parameters of the different VExUS grading systems assessed at ICU admission to predict acute kidney injury in 145 patients after cardiac surgery

Grading system	Grade	Specificity (CI)	Sensitivity (CI)	+LR (CI)	− LR (CI)
VExUS A	1	41% (31–51%)	73% (59–85)	1.24 (0.98–1.57)	0.65 (0.40–1.07)
	2	67% (56–76%)	55% (40–69%)	1.65 (1.13–2.42)	0.67 (0.49–0.93)
	3	86% (78–92%)	39% (26–54%)	2.86 (1.54–5.30)	0.71 (0.56–0.89)
VExUS B	1	41% (31–51%)	73% (59–85%)	1.24 (0.98–1.57)	0.65 (0.40–1.07)
	2	67% (56–76%)	55% (40–69%)	1.65 (1.13–2.42)	0.67 (0.49–0.93)
	3	77% (67–85%)	43% (29–58%)	1.87 (1.15–3.05)	0.74 (0.58–0.95)
VExUS C	1	41% (31–51%)	73% (59–85%)	1.24 (0.98–1.57)	0.65 (0.40–1.07)
	2	87% (78–92%)	39% (26–54%)	2.86 (1.55–5.30)	0.71 (0.56–0.89)
	3	96% (89–99%)	27% (15–41%)	6.37 (2.19–18.5)	0.77 (0.65–0.91)
VExUS D	2	52% (42–62%)	61% (46–74%)	1.28 (0.94–1.73)	0.74 (0.51–1.08)
	3	70% (59–79%)	45% (31–60%)	1.49 (0.96–2.29)	0.79 (0.61–1.03)
VExUS E	2	79% (69–87%)	41% (27–56%)	1.96 (1.17–3.28)	0.75 (0.59–0.95)
	3	93% (85–97%)	29% (17–43%)	3.92 (1.69–9.07)	0.77 (0.64–0.92)
Portal Doppler only	Mild	73% (64–82%)	39% (25–52%)	1.46 (0.90–2.37)	0.83 (0.65–1.08)
	Severe	91% (86–97%)	27% (14–39%)	3.12 (1.39–7.01)	0.80 (0.67–0.96)
Hepatic vein Doppler only	Mild	56% (46–66%)	51% (37–65%)	1.16 (0.81–1.66)	0.88 (0.62–1.23)
	Severe	84% (76–91%)	34% (20–48%)	2.11 (1.15–3.89)	0.79 (0.63–0.98)
Renal Doppler only	Mild	80% (72–88%)	45% (31–59%)	2.27 (1.36–3.77)	0.69 (0.52–0.90)
	Severe	94% (89–99%)	25% (12–37%)	3.92 (1.57–9.81)	0.81 (0.68–0.95)
CVP	≥ 8 mmHg	48% (37–59%)	77% (61–88%)	1.47 (1.13–1.90)	0.49 (0.28–0.86)
	≥ 10 mmHg	66% (55–75%)	58% (42–73%)	1.71 (1.16–2.51)	0.64 (0.44–0.91)
	≥ 12 mmHg	83% (73–90%)	33% (20–49%)	1.91 (1.02–3.59)	0.81 (0.66–1.01)

*CI* confidence intervals, *CVP* central venous pressure, *+LR* positive likelihood ratio, *− LR* negative likelihood ratio

**Fig. 4:**
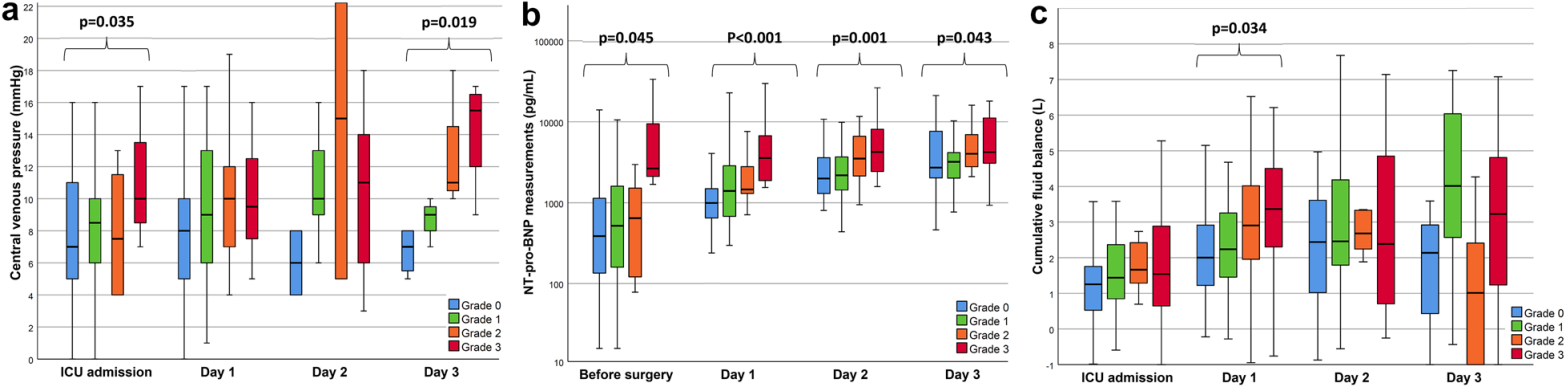
Clinical parameters in relationship with the Venous Excess UltraSound (VExUS) grading system C during the peri-operative period. **a** Central venous pressure at the time of ultrasound assessment in relationship with VExUS C grading system. **b** N-terminal pro-beta natriuretic peptide (NT-pro-BNP) in relationship with VExUS C grading system. **c** Cumulative fluid balance in relationship with VExUS C grading system. Significant results (*p* < 0.05) are highlighted. Complete results of comparisons are presented in Additional file 1: Table S3

Other congestion markers including cumulative fluid balance, NT-pro-BNP and CVP were associated with severe congestion (Grade 3) in all studied VExUS grading systems (see Additional file 1: Table S5). However, a statistical interaction with the time of assessment is present in multiple analyses. For the VExUS C grading system, severe congestion (Grade 3) was associated with a greater cumulative fluid balance (*β* = 899 CI 470; 1327 *p* < 0.001) and a higher CVP (*β* = 2.4 CI 0.70; 4.0 *p* = 0.004) without statistical interaction with the time of assessment. While an interaction was present for the analysis involving NT-pro-BNP, a significant difference in NT-pro-BNP measurements among grades was also present at each studied timepoint was as shown in Fig. 4. Comparison for other VExUS grading systems yielded similar results (see Additional file 1: Tables S6 to S10).

## Discussion

**Fig. 5:**
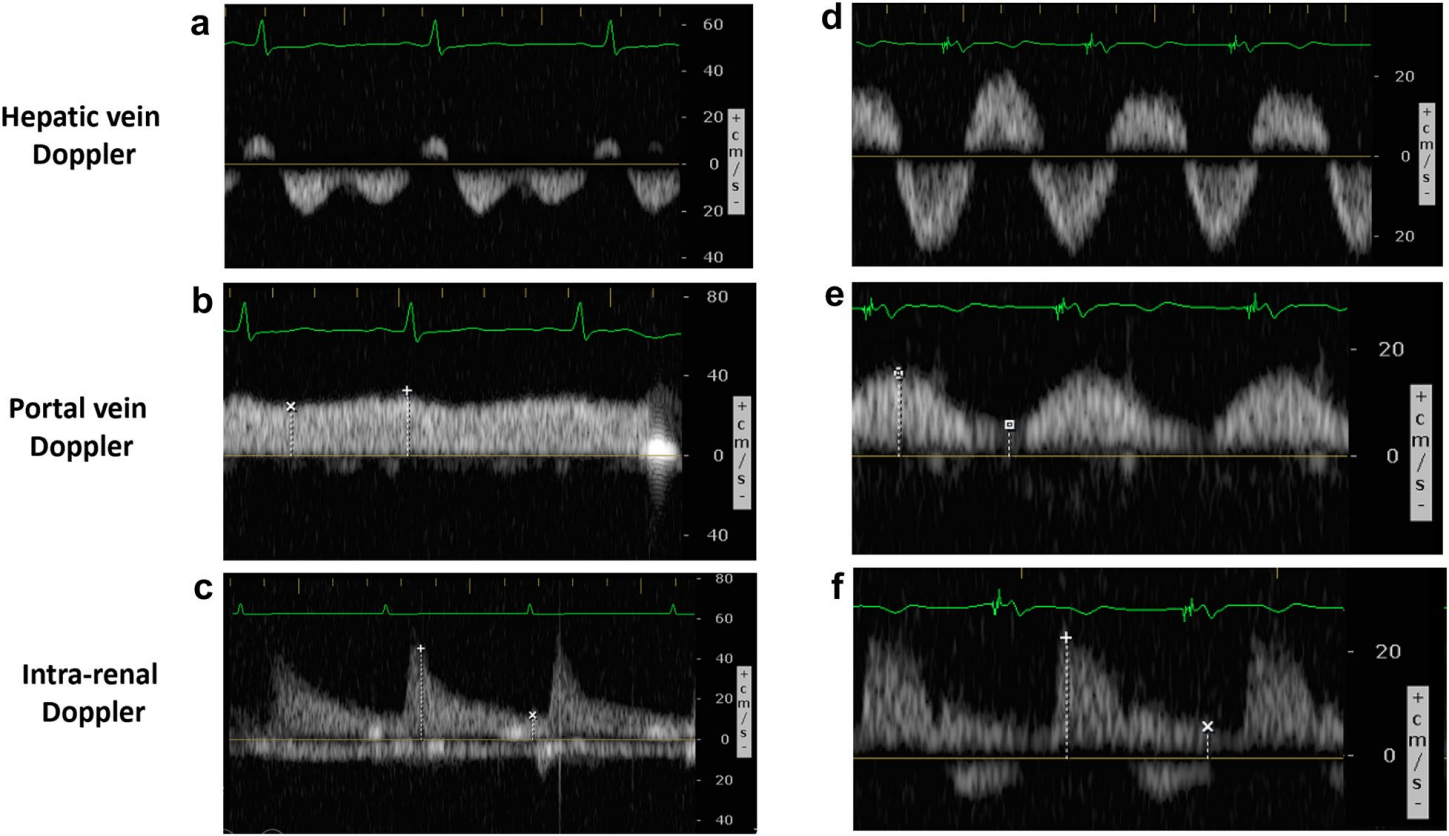
Example of VExUS C grading system assessment in cardiac surgery. Patient #1: A 55-year-old woman undergoing tricuspid valve repair aortic valve replacement and mitral valve replacement known with chronic kidney disease (baseline eGFR = 35 mL/min/1.73 m^2^) with left ventricular ejection fraction of 40% and a high risk of major complications (EuroSCORE II = 16.8%) presented the following ultrasound findings at ICU admission after surgery: **a** Normal hepatic triphasic pattern, **b** a non-pulsatile portal flow and **c** continuous intral-renal venous flow and an IVC diameter of > 2.1 cm (not shown) corresponding to Grade 1 of VExUS C grading system. The patient did not develop acute kidney injury, was extubated 2.2 h after ICU admission and was discharged from the ICU less than 24 h after surgery. Patient #2: A 70-year-old man undergoing mitral valve repair with a left ventricular ejection fraction of 50% and a moderate risk of major complication (EuroSCORE II = 1.54%) presented the following ultrasound findings at ICU admission after surgery complicated by right ventricular dysfunction after cardiopulmonary bypass: **d** Systolic reversal of the hepatic venous flow, **e** severe portal flow pulsatility and **f** severe alteration in intra-renal venous flow corresponding to Grade 3 of the VExUS C grading system. The patient developed severe acute kidney injury and delirium in the post-operative period

In this work, we aimed to investigate the performance of different venous congestion grading systems based on ultrasound markers to predict AKI after cardiac surgery. We found that severe congestion, defined as the presence of severe flow abnormalities in multiple Doppler patterns with a dilated IVC (VExUS grading system C—Grade 3), offered the strongest association with the development of subsequent AKI compared with other combinations of ultrasonographic features. Examples of VExUS C grading are presented in Fig. 5. Importantly, this association remained significant after adjustment with known risk factors for AKI as well as vasopressor–inotropic support after surgery, with a HR of 2.82. Furthermore, the presence of severe congestion defined by the VExUS C grading system at ICU admission after cardiac surgery offered a high specificity but low sensitivity to predict AKI resulting in a moderate +LR which outperformed the use of common CVP measurement cut-offs. Of note, IVC dilatation alone had poor diagnostic performance (Specificity 41%) suggesting that this commonly used ultrasound assessment in the ICU is not sufficient to detect clinically significant congestion. However, VExUS grading systems that included IVC assessment had small improvement in specificity suggesting that IVC dilatation might be a useful to avoid false-positives.

While there is now a widespread agreement that systemic venous hypertension resulting from fluid overload and right ventricular failure is associated with multiorgan injury and adverse outcomes [39]. POCUS may enable the clinician to detect clinically significant systemic venous hypertension. However, each of the proposed markers has some pitfalls and limitations. Hepatic vein Doppler is strongly influenced by tricuspid regurgitation which may influence its interpretation [40]. Pulsatile portal vein flow and IVC dilatation have been reported in healthy athletic volunteers which raise the possibility of false-positives [24, 41]. Finally, intra-renal venous Doppler is more technically challenging to perform and has a greater chance to provide ambiguous results in patients with poor ultrasound penetration or with devices offering less-sensitive Doppler capabilities. We therefore used a combination of these findings to better predict AKI and showed that it outperformed CVP measurement to predict congestive AKI. Previous studies have reported associations between echographic findings of right ventricular dysfunction and AKI after cardiac surgery [42, 43]. In addition to requiring advanced training, a high number of patients (> 25%) were excluded from these studies because of inadequate image quality. This raise concerns about the clinical usefulness of these assessments [42, 43]. Furthermore, in contrast to previous work, we performed a time-dependant analysis with repeated measurements. In addition, we performed multivariable adjustment for obvious potential confounders including validated scores summarizing the baseline risk of AKI and hemodynamic stability after cardiac surgery [30, 32].

In the present work, the low sensitivity to predict AKI is not surprising since venous congestion is unlikely to be a contributive factor in all cases. A multitude of other factors, venous congestion being only one of them, may trigger AKI in the peri-operative period [44]. However, the positive likelihood ratio exhibited by VExUS C Grade 3 indicates that most patients (96%) with these ultrasound features at ICU admission will develop AKI in the post-operative period. A small proportion of patients did not develop AKI despite presenting signs of severe venous congestion. Clearly, the presence of severe congestion alone is not enough to perfectly predict organ failure. However, this may not be entirely unexpected as perfusion also depends on arterial flow. Damman et al. elegantly demonstrated in a cohort of acutely decompensated congestive heart failure patients that venous hypertension was particularly deleterious in patients with reduced cardiac output [45]. This finding is also consistent with early animal experiments in which only very high venous pressure (≥ 25 mmHg) resulted in a decrease in the glomerular filtration rate when arterial blood flow was maintained [7, 46]. It is likely that the VExUS grading system, while being useful to assess the clinical importance of venous hypertension, will only give us partial information on how to guide intervention aimed at improving organ perfusion which could be complemented additional information related to arterial pefusion.

The present work has several limitations. First, we performed a relatively small single-center study including only cardiac surgery patients which limits the generalizability of the findings. Most importantly, the formation of interstitial edema also depends on vascular permeability which may be quite variable depending of the underlying disease. Consequently, caution should be taken when interpreting these finding in other clinical contexts. Furthermore, while the relationship between portal Doppler patterns and other echocardiographic variables during cardiac surgery has been previously described by our group [20] and others [47], we did not assess right ventricular function in this study which precludes us to determine if systolic right ventricular dysfunction was an important mechanism associated with severe VExUS grade in our cohort. Furthermore, because this analysis was based on retrospective data, other pertinent ultrasound features such as the respiratory collapsibility, 3-dimensional measurements of the IVC [48] or the evaluation of extra-vascular lung water could not be integrated in the VExUS grading systems. Finally, the confidence interval over the diagnostic performance parameters (sensitivity, specificity, +LR, − LR) is large due to the limited number of patients available in this cohort which also limits the power to detect significant differences in the performance of the studied grading systems. Consequently, we cannot definitely confirm based on the available data the superiority of the VExUS C grading system compared to the other candidate classifications or compared to individual findings.

## Conclusions

In conclusion, we used existing data to propose a novel grading system for venous congestion, the Venous Excess UltraSound (VExUS) grading system based on the combination of multiple ultrasound findings. The presence of at least two severe alterations of hepatic vein, portal vein or intra-renal venous flow on pulse-wave Doppler ultrasound with an IVC of ≥ 2 cm of diameter at ICU admission after cardiac surgery indicates a high risk of post-operative AKI. Further studies should aim to validate this grading system in different clinical settings, confirm the optimal criteria for diagnostic performance and determine whether it could be used to personalize interventions to improve organ perfusion.

## Supplementary information

**Additional file 1.** Quantifying systemic congestion with Point-Of-Care ultrasound: development of the Venous EXcess UltraSound (VExUS) grading system.

## Data Availability

The datasets used and/or analyzed during the current study are available from the corresponding author on reasonable request.

## Abbreviations

AKI: Acute kidney injury
CI: Confidence interval
CVP: Central venous pressure
EuroSCORE II: European System Operative Score Risk Evaluation score
HR: Hazard ratio
KDIGO: Kidney disease: improving global outcomes
ICU: Intensive care unit
IQR: Interquartile range
IVC: Inferior vena cava
LR: Likelihood ratio
LVEF: Left ventricular ejection fraction
NT-Pro-BNP: N-terminal pro-beta natriuretic peptide
PF: Pulsatility fraction
POCUS: Point-Of-Care ultrasound
SD: Standard deviation
VExUS: Venous Excess Ultrasound
VIS: Vasopressor–inotrope score
