# Supplementary material for: Quantifying systemic congestion with Point-Of-Care ultrasound: development of the venous excess ultrasound grading system
Source: Ultrasound J. 2020 Apr 9;12:16. doi: 10.1186/s13089-020-00163-w (PMC7142196; doi:10.1186/s13089-020-00163-w)
Supplement: Supplementary file 1 — Additional file 1. Quantifying systemic congestion with Point-Of-Care ultrasound: development of the Venous EXcess UltraSound (VExUS) grading system. [file 13089_2020_163_MOESM1_ESM.docx]

Additional materials: Quantifying systemic congestion with Point-Of-Care ultrasound: Development of the Venous EXcess UltraSound (VEXUS) score

eTable S1 : Baseline characteristics of studied patients

| **Total (n=145)** | | **Total**  **(n=145)** | **No AKI**  **(n=96)** | **AKI**  **(n=49)** | **p** |
| --- | --- | --- | --- | --- | --- |
| **Age (years)** | | 66 ±12.9 | 65 ±14 | 68 ±12 | 0.31 |
| **Female gender (n)** | | 38 (26.2%) | 30 (31.3%) | 8 (16.3%) | 0.05 |
| **Body mass index (Kg/m^2^)** | | 28.9 ±4.7 | 28.3 ±4.7 | 30.1 ±4.6 | 0.025 |
| **EuroSCORE II (%)** | | 2.96 (1.70; 4.79) | 2.74 (1.64; 4.07) | 3.70 (1.86; 7.60) | 0.01 |
| **Preoperative AKI risk score (%)** | | 22.9 (14.1; 35.3) | 20.6 (11.6; 31.0) | 28.2 (19.9; 48.6) | <0.001 |
| **eGFR (mL/Kg/1.73m^2^)** | | 75.9 ±20.3 | 76.3 ±19.6 | 74.2 ±21.8 | 0.47 |
| **Diabetes (n)** | | 50 (34.7%) | 30 (31.3%) | 20 (33.9%) | 0.25 |
| **Recent myocardial infarction (<90 days) (n)** | | 18 (12.5%) | 14 (14.6%) | 4 (8.2%) | 0.27 |
| **Left ventricular ejection fraction before surgery (%)** | | 55 (45; 60) | 55 (45; 60) | 55 (42; 60) | 0.70 |
| **Diuretic use before surgery (n)** | | 49 (33.8%) | 28 (29.2%) | 21 (42.9%) | 0.10 |
| **Type of surgery (n)** | Isolated CABG | 42 (29.0%) | 34 (35.4%) | 8 (16.3%) | 0.08 |
|  | One procedure other than CABG | 38 (26.2%) | 27 (28.1%) | 12 (24.5%) |  |
|  | 2 procedures | 50 (34.5%) | 28 (29.2%) | 22 (44.9%) |  |
|  | ≥3 procedures | 11 (7.6%) | 6 (6.3%) | 6 (12.2%) |  |
|  | Cardiac transplantation | 2 (1.4%) | 1 (1.0%) | 1 (2.0%) |  |
|  | Surgery on thoracic aorta | 10 (6.9%) | 4 (4.2%) | 6 (12.2%) | 0.07 |
| **Context of surgery (n)** | Elective | 97 (66.2%) | 67 (69.1%) | 31 (63.3%) | 0.72 |
|  | Urgent* | 48 (33.1%) | 30 (30.9%) | 18 (36.7%) |  |
| **NT-pro-BNP before surgery** | | 475 (155; 1588) | 390 (146; 1532) | 690 (172; 1922) | 0.16 |

*Patients who have not been electively admitted for operation but who require intervention or surgery on the current admission for medical reasons. These patients cannot be sent home without a definitive procedure. Legend: BMI: Body mass index, CABG: coronary artery bypass grafting, COPD: chronic pulmonary obstructive disorder, eGFR: estimated glomerular filtration rate calculated using the MDRD equation, HTN: chronic hypertension, LVEF: left ventricular ejection fraction.

eTable S2: Baseline clinical characteristics according to pre-operative VEXUS classification prototypes.

|  | | N | Euroscore II  (%) | | Pre- operative AKI  risk score (%) | | Age | | LVEF | | BMI | | Female  gender | | Diabetes | | CAD | |
| --- | --- | --- | --- | --- | --- | --- | --- | --- | --- | --- | --- | --- | --- | --- | --- | --- | --- | --- |
|  |  |  | Mean ±SD | p | Median (IQR) | p | Mean ±SD | p | Mean ±SD | p | Median (IQR) | p | N (%) | p | N (%) | p | N (%) | p |
| VEXUS  A | 0 | 66 | 3.77 ±3.36 | <0.001^a^ | 21.6 (13.6-32.2) | 0.01 | 67 ±11.8 | 0.45 | 53.6 ±12.1 | 0.6 | 29 (26-33) | 0.73 | 22 (33%) | 0.15 | 23 (35%) | 0.34 | 12 (18%) | 0.61 |
|  | 1 | 55 | 3.48 ±3.2 |  | 21.1 (11.7-29) |  | 63.9 ±12.1 |  | 50.6 ±13.7 |  | 28 (25-32) |  | 9 (16%) |  | 18 (33%) |  | 11 (20%) |  |
|  | 2 | 13 | 7.89 ±7.73 |  | 39.4 (32.6-51.3)* |  | 68.2 ±16.2 |  | 50.2 ±14.8 |  | 28 (26-31) |  | 3 (23%) |  | 7 (54%) |  | 4 (31%) |  |
|  | 3 | 11 | 8.65 ±7.25 |  | 24.2 (15.5-37) |  | 68.4 ±18.8 |  | 51.7 ±11.4 |  | 27 (25-30) |  | 4 (36%) |  | 2 (18%) |  | 1 (9%) |  |
| VEXUS  B | 0 | 66 | 3.77 ±3.36 | <0.001^b^ | 21.6 (13.6-32.2) | 0.02 | 67 ±11.8 | 0.17 | 53.6 ±12.1 | 0.38 | 29 (26-33) | 0.7 | 22 (33%) | 0.62 | 23 (35%) | 0.93 | 12 (18%) | 0.97 |
|  | 1 | 55 | 3.48 ±3.2 |  | 21.1 (11.7-29) |  | 63.9 ±12.1 |  | 50.6 ±13.7 |  | 28 (25-32) |  | 9 (16%) |  | 18 (33%) |  | 11 (20%) |  |
|  | 2 | 9 | 8.64 ±8.92 |  | 34.8 (24.2-49.8)* |  | 73.6 ±8.9 |  | 54.8 ±13.3 |  | 28 (27-32) |  | 2 (22%) |  | 4 (44%) |  | 2 (22%) |  |
|  | 3 | 15 | 7.99 ±6.59 |  | 35.3 (15.5-51.3) |  | 65.1 ±20 |  | 48.6 ±12.9 |  | 27 (25-30) |  | 5 (33%) |  | 5 (33%) |  | 3 (20%) |  |
| VEXUS  C | 0 | 66 | 3.77 ±3.36 | 0.011^c^ | 21.6 (13.6-32.2) | 0.50 | 67 ±11.8 | 0.22 | 53.6 ±12.1 | 0.57 | 29 (26-33) | 0.73 | 22 (33%) | 0.06 | 23 (35%) | 0.74 | 12 (18%) | 0.39 |
|  | 1 | 68 | 4.32 ±4.69 |  | 23.2 (14.9-40.4) |  | 64.7 ±13 |  | 50.5 ±13.8 |  | 28 (25-32) |  | 12 (18%) |  | 25 (37%) |  | 15 (22%) |  |
|  | 2 | 8 | 8.65 ±8.37* |  | 23.6 (16.9-35.3) |  | 64.3 ±20.2 |  | 50.9 ±13.3 |  | 27.5 (26-29) |  | 2 (25%) |  | 2 (25%) |  | 0 (0%) |  |
|  | 3 | 3 | 8.63 ±4.22 |  | 35.3 (15.5-60.6) |  | 79.3 ±9.1 |  | 54 ±5.3 |  | 27 (24-33) |  | 2 (67%) |  | 0 (0%) |  | 1 (33%) |  |
| VEXUS  D | 0 | 116 | 3.6 ±3.27 | <0.001^d^ | 21.8 (13.5-32) | 0.09 | 65.7 ±12.2 | 0.52 | 52.3 ±13.1 | 0.59 | 28 (26-33) | 0.55 | 29 (25%) | 0.54 | 39 (34%) | 0.62 | 23 (20%) | 1.00 |
|  | 1 | 13 | 7.16 ±7.92 |  | 24.2 (17.2-48.6) |  | 69.9 ±10.2 |  | 53.3 ±12.4 |  | 28 (26-31) |  | 3 (23%) |  | 6 (46%) |  | 2 (15%) |  |
|  | 2 | 16 | 7.92 ±6.37 |  | 34.5 (17.4-45.3) |  | 65.2 ±19.4 |  | 49 ±12.5 |  | 27.5 (25.5-30.5) |  | 6 (38%) |  | 5 (31%) |  | 3 (19%) |  |
| VEXUS  E | 0 | 133 | 4.07 ±4.1 | 0.012 | 22.7 (13.6-34.8) | 0.59 | 65.9 ±12.5 | 0.17 | 52.1 ±13.1 | 0.88 | 28 (26-32) | 0.72 | 34 (26%) | 0.26 | 47 (35%) | 0.68 | 27 (20%) | 0.21 |
|  | 1 | 9 | 7.89 ±8.15 |  | 22.9 (14.6-33.7) |  | 63.3 ±19.1 |  | 50.2 ±12.6 |  | 28 (27-29) |  | 2 (22%) |  | 3 (33%) |  | 0 (0%) |  |
|  | 2 | 3 | 8.63 ±4.22 |  | 35.3 (15.5-60.6) |  | 79.3 ±9.1 |  | 54 ±5.3 |  | 27 (24-33) |  | 2 (67%) |  | 0 (0%) |  | 1 (33%) |  |

Significant post-hoc pairwise comparisons after Bonferroni correction: ^a^ Grade 0 vs 2: p=0.01, Grade 0 vs 3: p=0.003, Grade 1 vs 2: p=0.006, Grade 1 vs 3: 0.002 ^b^ Grade 0 vs 2: p=0.009, Grade 0 vs 3: p=0.004, Grade 1 vs 2: p=0.005, Grade 1 vs 3: p=0.002 ^c^ Grade 0 vs 2: p=0.02 ^d^ Grade 0 vs 1: p=0.015, Grade 0 vs 2: p=0.001. Legend: AKI: acute kidney injury, BMI: Body mass index, CAD: coronary artery disease, LVEF: left ventricular ejection fraction.

eTable S3: Supplementary multivariable Proportional Hazards Models to predict acute kidney injury in 145 patients after cardiac surgery using the Venous EXcess UltraSound (VExUS) C grading system.

|  | Crude HR (CI) p-value | Adjusted HR (CI) p-value |
| --- | --- | --- |
| CBP duration (per h) | 1.24 (0.90; 1.74) 0.20 |  |
| Cardiac output at the end of cardiac surgery (L/min) |  | 1.18 (0.86; 1.62) p=0.30 |
| VExUS C Pattern 0 | Reference | Reference |
| VExUS C Pattern 1 | 1.20 (0.57; 2.51) p=0.64 | 0.98 (0.42; 2.28) p=0.96 |
| VExUS C Pattern 2 | 1.84 (0.69; 4.93) p=0.22 | 1.11 (0.35; 3.50) p=0.86 |
| VExUS C Pattern 3 | 3.90 (1.76; 8.66) p=0.001 | 3.35 (1.35; 8.25) p=0.009 |

Legend: CBP: cardiopulmonary bypass

eTable S4: McNemar test results to assess the presence of differences in specificity between VExUS C pattern 3 and other studied variables

| **VEXUS C pattern 3 compared to:** | **p-value** |
| --- | --- |
| CVP ≥8 mmHg | <0.001 |
| CVP ≥10 mmHg | <0.001 |
| CVP ≥12 mmHg | 0.004 |
| IVC >2 cm | <0.001 |
| VEXUS C pattern 2 | 0.04 |
| VEXUS C pattern 1 | <0.001 |
| VEXUS A pattern 3 | 0.04 |
| VEXUS B pattern 3 | <0.001 |
| VEXUS D pattern 3 | <0.001 |
| VEXUS E pattern 3 | 0.25 |
| Portal vein pulsatility Mild | <0.001 |
| Portal vein pulsatility Severe | 0.11 |
| Hepatic vein pattern Mild | <0.001 |
| Hepatic vein pattern Severe | 0.001 |
| Intra-renal venous pattern Mild | <0.001 |
| Intra-renal venous pattern Severe | 0.63 |

Legend: CVP: central venous pressure, IVC: inferior vena cava.

eTable S5: Association between VEXUS classification prototypes and commonly used clinical markers of venous congestion.

|  |  | **Cumulative fluid balance**  **(mL)** | | **NT-pro-BNP**  **(1 log)** | | **CVP**  **(mmHg)** | | **Vasoactive-inotropic score**  **(points)** | |
| --- | --- | --- | --- | --- | --- | --- | --- | --- | --- |
|  | **Grade** | **B (CI)** | **p** | **B (CI)** | **p** | **B (CI)** | **p** | **B (CI)** | **p** |
| **VEXUS A** | 0 | Reference category | | | | | | | |
|  | 1 | 172 (-170; 513)* | 0.32 | 0.072 (-0.041; 0.184) | 0.21 | 0.0 (-1.4; 1.4) | 0.99 | 0.6 (-1.2; 2.4)* | 0.49 |
|  | 2 | 387 (33; 741) | 0.03 | 0.157 (0.058; 0.256)* | 0.002 | 1.2 (-0.2; 2.6)* | 0.09 | 1.0 (-0.4; 2.5) | 0.17 |
|  | 3 | 731 (366; 1095) | <0.001 | 0.220 (0.112; 0.329) | <0.001 | 1.9 (0.5; 3.2)* | 0.009 | 1.5 (-0.8; 3.0) | 0.06 |
| **VEXUS B** | 0 | Reference category | | | | | | | |
|  | 1 | 160 (-183; 503)* | 0.36 | 0.070 (-0.043; 0.182) | 0.23 | 0.0 (-1.4; 1.4) | 0.99 | 0.6 (-1.2; 2.4)* | 0.52 |
|  | 2 | 325 (-51; 701) | 0.09 | 0.137 (0.025; 0.249) | 0.02 | 1.0 (-0.6; 2.7) | 0.22 | 0.4 (-1.1; 2.0) | 0.60 |
|  | 3 | 718 (364; 1072) | <0.001 | 0.206 (0.107; 0.304) | <0.001 | 1.8 (0.5; 3.0)* | 0.007 | 1.7 (0.3; 3.1) | 0.02 |
| **VEXUS C** | 0 | Reference category | | | | | | | |
|  | 1 | 271 (-25; 567) | 0.07 | 0.126 (0.034; 0.218) | 0.007 | 0.6 (-0.6; 1.8) | 0.34 | 0.8 (-0.5; 2.2)* | 0.23 |
|  | 2 | 534 (134; 934)* | 0.009 | 0.181 (0.059; 0.304) | 0.004 | 1.1 (-0.7; 2.8)* | 0.23 | 1.0 (-0.8; 2.8) | 0.28 |
|  | 3 | 899 (470; 1327) | <0.001 | 0.250 (0.124; 0.376)* | <0.001 | 2.4 (0.7; 4.0) | 0.004 | 1.9 (-0.1; 3.9) | 0.07 |
| **VEXUS D** | 1 | Reference category | | | | | | | |
|  | 2 | 119 (-197; 434)* | 0.46 | 0.127 (0.039; 0.215) | 0.005 | 0.5 (-0.8; 1.9) | 0.45 | -0.1 (-1.5; 1.4) | 0.93 |
|  | 3 | 395 (63; 726)* | 0.02 | 0.254 (0.167; 0.341)* | <0.001 | 1.7 (0.5; 2.9)* | 0.005 | 1.6 (0.04; 3.3) | 0.049 |
| **VEXUS E** | 1 | Reference category | | | | | | | |
|  | 2 | 206 (-111; 523)* | 0.20 | 0.096 (0.002; 0.191) | 0.05 | 0.4 (-1.0; 1.8) | 0.58 | 0.5 (-1.3; 2.3) | 0.58 |
|  | 3 | 436 (89; 783) | 0.014 | 0.205 (0.103; 0.307)* | <0.001 | 1.9 (0.5; 3.3) | 0.007 | 1.3 (-0.6; 3.3) | 0.17 |

Generalized estimating equation models with the time of assessment included in the models as a covariable. Results are shown in ß estimates representing the estimated variation of the parameter for each VEXUS grade compared to grade 0 with 95% confidence intervals (CI). ***Denotes that a significant interaction with the time of assessment was present.** NT-Pro-BNP: N-terminal-Pro-Beta natriuretic peptide; CVP: central venous pressure; systolic pulmonary artery pressure.

eTable S6: Commonly used parameters of congestion in relationship with the VEXUS C classification in the peri-operative period.

| **Timepoint** | **Grade 0** | **Grade 1** | **Grade 2** | **Grade 3** | **p** |
| --- | --- | --- | --- | --- | --- |
| **Fluid Balance (L)** | | | | | |
| **ICU admission** | 1.0 (0.5; 1.6) | 1.3 (0.9; 1.8) | 1.3 (0.5; 1.6) | 1.4 (0.3; 1.7) | 0.38 |
| **Day 1** | 1.3 (0.7; 1.9) | 1.3 (0.8; 2.1) | 1.8 (0.8; 2.9) | 2.5 (1.5; 2.8) | 0.034 |
| **Day 2** | 2.0 (1.2; 3.2) | 2.2 (1.5; 3.3) | 2.6 (1.5; 3.4) | 2.9 (1.9; 3.7) | 0.19 |
| **Day 3** | 2.0 (0.6; 3.3) | 2.8 (1.9; 5.2) | 2.2 (1.2; 4.3) | 3.3 (0.9; 5.0) | 0.11 |
| **Central Venous Pressure (mmHg)** | | | | | |
| **ICU admission** | 8.2 ±4.0 | 8.5 ±3.4 | 7.9 ±3.5 | 11.1 ±3.1 | 0.035^a^ |
| **Day 1** | 8.3 ±5.0 | 9.2 ±4.5 | 10.2 ±4.3 | 10.0 ±3.5 | 0.54 |
| **Day 2** | 6.0 ±2.8 | 10.8 ±4.0 | 15.0 ±14.1 | 10.4 ± 5.2 | 0.42 |
| **Day 3** | 6.8 ±1.5 | 8.7 ±1.5 | 13.0 ±4.4 | 14.3 ±3.6 | 0.019^b^ |
| **NT-pro-BNP (pg/mL)** | | | | | |
| **Before surgery** | 388 (135; 1137) | 520 (160; 1604) | 750 (121; 1521) | 2651 (1689; 33534) | 0.045^c^ |
| **Day1** | 997 (650; 1496) | 1401 (682; 2862) | 1464 (1287; 3138) | 3570 (1881; 8608) | <0.001^d^ |
| **Day2** | 1995 (1304; 3607) | 2183 (1401; 3851) | 3514 (1919; 6894) | 4255 (2445; 8354) | 0.001^e^ |
| **Day 3** | 2721 (2031; 7643) | 3212 (2018; 4167) | 4042 (2789; 6945) | 4223 (3081; 11149) | 0.043 |
| **Vasoactive-inotropic score (points)** | | | | | |
| **ICU admission** | 0 (0; 5) | 5 (0; 13) | 1 (0; 7) | 6 (2; 15) | 0.005^f^ |
| **Day1** | 0 (0; 3) | 0 (0; 3) | 0 (0;10) | 6 (0; 20) | 0.008^g^ |
| **Day2** | 0 (0; 0) | 0 (0; 0) | 0 (0; 0) | 0 (0; 6) | 0.09 |
| **Day 3** | 0 (0; 3) | 0 (0; 3) | 0 (0;10) | 0 (0; 0) | 0.26 |

Significant post-hoc pairwise comparisons after Bonferroni correction: ^a^ Grade 0 vs 3: p=0.037, ^b^ Grade 0 vs 3: p=0.019, ^c^ Grade 0 vs 3: p=0.03, ^d^ Grade 0 vs 3: p<0.001, ^e^ Grade 0 vs 3: p=0.002. ^f^ Grade 0 vs 1: p=0.014. ^g^ Grade 0 vs 2: p=0.036, Grade 0 vs 3: p=0.009.

Legend: NT-Pro-BNP: N-terminal-Pro-Beta natriuretic peptide; ICU: intensive care unit

eTable S7: Commonly used parameters of congestion in relationship with the VEXUS A classification in the peri-operative period.

| **Timepoint** | **Grade 0** | **Grade 1** | **Grade 2** | **Grade 3** | **p** |
| --- | --- | --- | --- | --- | --- |
| **Fluid balance (L)** | | | | | |
| **ICU admission** | 1.0 (0.5; 1.6) | 1.5 (0.9; 1.8) | 1.2 (0.8; 1.8) | 1.4 (0.5; 1.7) | 0.28 |
| **Day 1** | 1.3 (0.7; 1.9) | 1.2 (0.7; 2.1) | 1.4 (1.0; 2.1) | 2.0 (1.2; 2.9) | 0.035 |
| **Day 2** | 2.0 (1.2; 3.2) | 1.6 (1.2; 2.8) | 2.5 (1.7; 3.3) | 2.7 (1.8; 3.7) | 0.08 |
| **Day 3** | 2.0 (0.6; 3.3) | 2.5 (1.9; 3.3) | 3.0 (1.9; 5.8) | 2.4 (1.2; 4.3) | 0.1 |
| **Central venous pressure (mmHg)** | | | | | |
| **ICU admission** | 8.2 ±4.0 | 8.5 ±3.4 | 8.4 ±3.3 | 9.8 ±3.6 | 0.36 |
| **Day 1** | 8.2 ±5.0 | 6.7 ±3.8 | 11.5 ±3.9 | 10.1 ±3.9 | 0.010^a^ |
| **Day 2** | 6.0 ±2.8 | 9.3 ±1.7 | 11.4 ±4.6 | 11.3 ±6.8 | 0.019 |
| **Day 3** | 6.8 ±1.5 | 10 | 8.0 ±1.4 | 13.7 ±3.6 | 0.049 |
| **NT-pro-BNP (pg/mL)** | | | | | |
| **Before surgery** | 388 (135; 1137) | 383 (138; 1270) | 1682 (520; 2792) | 1521 (369;2651) | 0.005^b^ |
| **Day1** | 997 (650; 1496) | 903 (608; 2127) | 2275 (988; 3914) | 2458 (1464; 4928) | <0.001^c^ |
| **Day2** | 1995 (1304; 3607) | 1961 (1343; 2608) | 2525 (1513; 4196) | 3554 (2278; 6894) | 0.001^d^ |
| **Day 3** | 2721 (2031; 7643) | 2594 (1785; 3486) | 3459 (2481; 4314) | 4223 (2842; 9117) | 0.006^e^ |
| **Vasoactive-inotropic score (points)** | | | | | |
| **ICU admission** | 0 (0; 5) | 2 (0; 11) | 5 (0; 16) | 3 (0; 8) | 0.02^f^ |
| **Day 1** | 0 (0; 3) | 0 (0; 0) | 3 (0; 4) | 4 (0; 13) | <0.001^g^ |
| **Day 2** | 0 (0; 0) | 0 (0; 0) | 0 (0; 0) | 0 (0; 2) | 0.49 |
| **Day 3** | 0 (0; 0) | 0 (0; 0) | 0 (0; 0) | 0 (0; 0) | 0.19 |

Significant post-hoc pairwise comparisons after Bonferroni correction: ^a^ Grade 1 vs 2: p=0.016, ^b^ Grade 0 vs 2: p=0.025, ^c^ Grade 0 vs 2: p=0.02, Grade 0 vs 3: p<0.001, Grade 1 vs 3: p=0.011, ^d^ Grade 0 vs 3: p=0.004, Grade 1 vs 3: p=0.009, Grade 2 vs 3: p=0.028, ^e^ Grade 1 vs 3: p=0.003. ^f^ Grade 0 vs 2: p=0.026. ^g^ Grade 1 vs 2 : p=0.014, Grade 1 vs 3: p<0.001.Legend: NT-Pro-BNP: N-terminal-Pro-Beta natriuretic peptide; ICU: intensive care unit

eTable S8: Commonly used parameters of congestion in relationship with the VEXUS B classification in the peri-operative period.

| **Timepoint** | **Grade 0** | **Grade 1** | **Grade 2** | **Grade 3** | **p** |
| --- | --- | --- | --- | --- | --- |
| **Fluid balance (L)** | | | | | |
| **ICU admission** | 1.0 (0.5;1.6) | 1.5 (0.9; 1.8) | 1.0 (0.5; 1.5) | 1.3 (0.6; 1.8) | 0.19 |
| **Day 1** | 1.3 (0.7; 1.9) | 1.2 (0.7; 2.1) | 1.6 (0.8; 2.1) | 1.7 (1.2; 2.8) | 0.06 |
| **Day 2** | 2.0 (1.2; 3.2) | 1.6 (1.2; 2.8) | 2.5 (1.7; 3.0) | 2.7 (1.8; 3.6) | 0.08 |
| **Day 3** | 2.0 (0.6; 3.3) | 2.5 (1.9; 3.3) | 4.2 (3.0; 5.6) | 2.4 (1.2; 4.9) | 0.037 |
| **Central venous pressure (mmHg)** | | | | | |
| **ICU admission** | 8.2 ±4.0 | 8.5 ±3.4 | 8.9 ±3.8 | 9.2 ±3.4 | 0.034 |
| **Day 1** | 8.2 ±5.0 | 6.7 ±3.8 | 10.8 ±4.8 | 10.6 ±3.5 | 0.017 |
| **Day 2** | 6.0 ±2.8 | 9.3 ±1.7 | 7.5 ±2.1 | 11.8 ±5.9 | 0.012 |
| **Day 3** | 6.8 ±1.5 | 10 | 9 | 12.9 ±4.1 | 0.18 |
| **NT-pro-BNP (pg/mL)** | | | | | |
| **Before surgery** | 388 (135; 1137) | 383 (138; 1270) | 1682 (520; 4021) | 1521 (377; 2699) | 0.005 |
| **Day1** | 997 (650; 1496) | 903 (608; 2127) | 1236 (618; 2331) | 3070 (1850; 4928) | <0.001 |
| **Day2** | 1995 (1304; 3607) | 1961 (1343; 2608) | 2183 (1401; 3494) | 3361 (2211; 6296) | 0.011 |
| **Day 3** | 2721 (2031; 7643) | 2594 (1785; 3486) | 3380 (2641; 4234) | 3885 (2816; 7108) | 0.017 |
| **Vasoactive-inotropic score** | | | | | |
| **ICU admission** | 0 (0; 5) | 2 (0; 11) | 3 (0; 10) | 5 (0; 16) | 0.027^f^ |
| **Day 1** | 0 (0; 3) | 0 (0; 0) | 0 (0; 3) | 4 (0; 11) | <0.001^g^ |
| **Day 2** | 0 (0; 0) | 0 (0; 0) | 0 (0; 0) | 0 (0; 2) | 0.21 |
| **Day 3** | 0 (0; 0) | 0 (0; 0) | 0 (0; 0) | 0 (0; 0) | 0.36 |

Significant post-hoc pairwise comparisons after Bonferroni correction: ^a^ Grade 1 vs 2: p=0.016, ^b^ Grade 0 vs 2: p=0.025, ^c^ Grade 0 vs 2: p=0.02, Grade 0 vs 3: p<0.001, Grade 1 vs 3: p=0.011, ^d^ Grade 0 vs 3: p=0.004, Grade 1 vs 3: p=0.009, Grade 2 vs 3: p=0.028, ^e^ Grade 1 vs 3: p=0.003. ^f^ Grade 0 vs 3 p=0.035. ^g^ Grade 0 vs 3 : p=0.018, Grade 1 vs 3: p<0.001. Legend: NT-Pro-BNP: N-terminal-Pro-Beta natriuretic peptide; ICU: intensive care unit

eTable S9: Commonly used parameters of congestion in relationship with the VEXUS D classification in the peri-operative period.

| **Timepoint** | **Grade 1** | **Grade 2** | **Grade 3** | **p** |
| --- | --- | --- | --- | --- |
| **Fluid Balance (L)** | | | | |
| **ICU admission** | 1.1 (0.7; 1.8) | 0.9 (0.4; 1.4) | 1.3 (0.6; 1.8) | 0.21 |
| **Day 1** | 1.3 (0.9; 2.1) | 1.2 (0.5; 2.1) | 1.6 (0.8; 2.7) | 0.12 |
| **Day 2** | 1.6 (1.0; 2.8) | 2.4 (1.5; 2.9) | 2.7 (1.8; 3.6) | 0.009^a^ |
| **Day 3** | 2.3 (0.7; 3.0) | 3.3 (1.5; 5.2) | 2.4 (1.7; 4.2) | 0.27 |
| **Central venous pressure (mmHg)** | | | | |
| **ICU admission** | 8.3 ±3.8 | 8.5 ±3.9 | 9.3 ±3.4 | 0.013^b^ |
| **Day 1** | 7.3 ±4.8 | 9.8 ±4.7 | 10.5 ±3.5 | 0.012^c^ |
| **Day 2** | 8.2 ±2.8 | 7.5 ±2.1 | 11.6 ±5.8 | 0.002^d^ |
| **Day 3** | 7.0 ±2.6 | 8.5 ±0.7 | 12.3 ±4.2 | 0.08 |
| **NT-pro-BNP (pg/mL)** | | | | |
| **Before surgery** | 372 (134; 1137) | 1337 (484; 2934) | 1326 (434; 2675) | 0.002^e^ |
| **Day1** | 847 (589; 1483) | 1172 (711; 2205) | 2946 (1650; 4892) | <0.001^f^ |
| **Day2** | 1699 (1215; 2522) | 2139 (1378; 3414) | 3479 (2211; 6296) | <0.001^g^ |
| **Day 3** | 2094 (1470; 3108) | 3212 (2222; 4201) | 4163 (2848; 8026) | <0.001^h^ |
| **Vasoactive-inotropic score** | | | | |
| **ICU admission** | 1 (0; 8) | 2 (0; 6) | 4 (0; 16) | 0.31 |
| **Day 1** | 0 (0; 0) | 0 (0; 3) | 4 (0; 12) | <0.001^i^ |
| **Day 2** | 0 (0; 0) | 0 (0; 0) | 0 (0; 2) | 0.01^j^ |
| **Day 3** | 0 (0; 0) | 0 (0; 0) | 0 (0; 0) | 0.23 |

Significant post-hoc pairwise comparisons after Bonferroni correction: ^a^ Grade 1 vs 3: p=0.08, ^b^ Grade 1 vs 3: p=0.012, ^c^ Grade 1 vs 3: p=0.013, ^d^ Grade 1 vs 3: p=0.002, ^e^ Grade 0 vs 2: p<0.001, ^f^ Grade 0 vs 2: p<0.001, Grade 1 vs 2: p<0.001, ^g^ Grade 0 vs 2: p<0.001, Grade 1 vs 2: p=0.041, ^h^ Grade 0 vs 2: p<0.001, Grade 1 vs 2: p=0.007. ^I^ Grade 0 vs 2 : p<0.001, Grade 1 vs 2: p=0.007. ^j^ Grade 1 vs 2 : p=0.021.Legend: NT-Pro-BNP: N-terminal-Pro-Beta natriuretic peptide; ICU: intensive care unit

eTable S10: Commonly used parameters of congestion in relationship with the VEXUS E classification in the peri-operative period.

| **Timepoint** | **Grade 1** | **Grade 2** | **Grade 3** | **p** |
| --- | --- | --- | --- | --- |
| **Fluid balance (L)** | | | | |
| **ICU admission** | 1.1 (0.7; 1.8) | 1.4 (0.5; 2.1) | 1.3 (0.3; 1.7) | 0.91 |
| **Day 1** | 1.3 (0.8; 2.1) | 1.8 (0.6; 2.8) | 1.8 (1.4; 2.8) | 0.042 |
| **Day 2** | 2.1 (1.4; 3.2) | 2.5 (1.3; 3.7) | 2.9 (1.9; 3.7) | 0.052 |
| **Day 3** | 2.3 (1.3; 3.3) | 2.3 (1.9; 3.0) | 2.4 (1.7; 3.5) | 0.66 |
| **Central venous pressure (mmHg)** | | | | |
| **ICU admission** | 8.3 ±3.7 | 8.1 ±3.6 | 11.0 ±3.0 | 0.008^a^ |
| **Day 1** | 8.6 ±4.8 | 9.9 ±4.1 | 10.3 ±3.5 | 0.98 |
| **Day 2** | 10.3 ±4.3 | 15.0 ±14.1 | 10.1 ±4.9 | 0.61 |
| **Day 3** | 7.4 ±2.1 | 11.8 ±4.3 | 13.0 ± 4.2 | 0.22 |
| **NT-pro-BNP (pg/mL)** | | | | |
| **Before surgery** | 474 (150; 1533) | 369 (121; 1521) | 2651 (1689; 33534) | 0.023^b^ |
| **Day1** | 1159 (650; 2295) | 1475 (1287; 2891) | 3570 (1881; 7502) | <0.001^c^ |
| **Day2** | 2035 (1343; 3528) | 3530 (1919; 6362) | 4255 (2582; 8870) | <0.001^d^ |
| **Day 3** | 2955 (2018; 4102) | 4287 (3476; 7742) | 5121 (3291; 12583) | <0.001^e^ |
| **Vasoactive-inotropic score** | | | | |
| **ICU admission** | 2 (0; 8) | 1 (0; 7) | 5 (0; 15) | 0.32 |
| **Day 1** | 0 (0; 3) | 3 (0; 10) | 6 (0; 17) | <0.001^f^ |
| **Day 2** | 0 (0; 0) | 0 (0; 0) | 0 (0; 6) | 0.026^g^ |
| **Day 3** | 0 (0; 0) | 0 (0; 0) | 0 (0; 0) | 0.08 |

Significant post-hoc pairwise comparisons after Bonferroni correction: ^a^ Grade 1 vs 3: p=0.008, ^b^ Grade 0 vs 2: p=0.019, ^c^ Grade 0 vs 2: p<0.001, ^d^ Grade 0 vs 2: p<0.001, ^e^ Grade 0 vs 1: p=0.009, Grade 0 vs 2: p=0.001. ^f^Grade 0 vs 2: p=0.001. ^g^Grade 0 vs 2 : p=0.024. Legend: NT-Pro-BNP: N-terminal-Pro-Beta natriuretic peptide; ICU: intensive care unit
